# *Streptomyces misionensis* PESB-25 Produces a Thermoacidophilic Endoglucanase Using Sugarcane Bagasse and Corn Steep Liquor as the Sole Organic Substrates

**DOI:** 10.1155/2013/584207

**Published:** 2013-03-24

**Authors:** Marcella Novaes Franco-Cirigliano, Raquel de Carvalho Rezende, Mônica Pires Gravina-Oliveira, Pedro Henrique Freitas Pereira, Rodrigo Pires do Nascimento, Elba Pinto da Silva Bon, Andrew Macrae, Rosalie Reed Rodrigues Coelho

**Affiliations:** ^1^Departamento de Microbiologia Geral, Instituto de Microbiologia Paulo de Góes, Centro de Ciências da Saúde (CCS), Universidade Federal do Rio de Janeiro (UFRJ), Avenida Carlos Chagas Filho 373, Bloco I, Laboratório 055, 21941-902 Rio de Janeiro, RJ, Brazil; ^2^Departamento de Engenharia Bioquímica, Escola de Química, Centro de Tecnologia (CT), Universidade Federal do Rio de Janeiro (UFRJ), Avenida Athos da Silveira Ramos 149, Bloco E, sala 203, 21941-909 Rio de Janeiro, RJ, Brazil; ^3^Departamento de Bioquímica, Instituto de Química, Centro de Ciências Matemáticas e Natureza (CCMN), Universidade Federal do Rio de Janeiro (UFRJ), Avenida Athos da Silveira Ramos 149, Bloco A, sala 539, 21941-909 Rio de Janeiro, RJ, Brazil

## Abstract

*Streptomyces misionensis* strain PESB-25 was screened and selected for its ability to secrete cellulases. Cells were grown in a liquid medium containing sugarcane bagasse (SCB) as carbon source and corn steep liquor (CSL) as nitrogen source, whose concentrations were optimized using response surface methodology (RSM). A peak of endoglucanase accumulation (1.01 U*·*mL^−1^) was observed in a medium with SCB 1.0% (w/v) and CSL 1.2% (w/v) within three days of cultivation. *S. misionensis* PESB-25 endoglucanase activity was thermoacidophilic with optimum pH and temperature range of 3.0 to 3.6 and 62° to 70°C, respectively. In these conditions, values of 1.54 U mL^−1^ of endoglucanase activity were observed. Moreover, Mn^2+^ was demonstrated to have a hyperactivating effect on the enzyme. In the presence of MnSO_4_ (8 mM), the enzyme activity increased threefold, up to 4.34 U*·*mL^−1^. Mn^2+^ also improved endoglucanase stability as the catalyst retained almost full activity upon incubation at 50°C for 4 h, while in the absence of Mn^2+^, enzyme activity decreased by 50% in this same period. Three protein bands with endoglucanase activity and apparent molecular masses of 12, 48.5 and 119.5 kDa were detected by zymogram.

## 1. Introduction

Enzymatic hydrolysis of cellulose is a challenge worldwide, because currently we lack inexpensive and efficient enzymes to hydrolyse the 1.5 trillion tons of cellulose produced annually [1]. Enzyme blends and optimization are required to speed up enzymatic hydrolysis to make the process commercially viable. Cellulose is a homopolymer of *β*-1,4 linked glucose units presenting both amorphous and crystalline regions. Its hydrolysis is carried out by endo-*β*-1,4-glucanase (EC 3.2.1.4), which cleaves internal *β*-1,4-glycosidic bonds at random positions and forms insoluble reducing sugars, and by exo-*β*-1,4-glucanase (EC 3.2.1.91) that hydrolyses cellulose from its reducing and nonreducing ends releasing soluble reducing sugars with prevalence of cellobiose. The enzyme *β*-glucosidase (EC 3.2.1.21) converts cellobiose into glucose monomers [2]. An increase in the formation of free reducing and nonreducing ends from endo-acting cellulases could speed up the action of the exoglucanases and the total cellulose hydrolysis process. A significant amount of research on new endoglucanases has been done [3, 4]. 

 Cellulolytic organisms are ubiquitous in nature. They are mostly bacteria and fungi, aerobic or anaerobic, and mesophilic or thermophilic. Actinomycetes, which are Gram-positive filamentous soil bacteria, are well known for their ability to decompose complex molecules, particularly the lignocellulose components, which make them important agents in decomposition processes [5]. They have also been shown to produce thermostable cellulases, with alkalophilic and acidophilic characteristics [6, 7]. The work that has been done in our laboratory with strains from the *Streptomyces* genus indicates that endoglucanase activity is predominant in these bacterial cellulases. In previous studies from our laboratory, we reported that the culture supernatant of *S. malaysiensis* AMT-3, *S*. *drozdowiczii* M-7A, and *S*. *viridobrunneus* SCPE-09 presented endoglucanase activity with optimal pH in the range of 4.0 to 5.0, optimal temperature around 50°C and molecular masses, according to zymogram analyses, in the range of 37 to 178 kDa [8–10].

This study investigated cellulase production by an actinobacterial strain, *S. misionensis* PESB-25. Experimental design was performed to optimize endo-*β*-1,4-glucanase production using SCB as the main carbon source and CSL as nitrogen source. As seen before, these low-cost materials can be suitable for cellulases production [8–10]. The electrophoretic profiles of extracted enzymes were determined by zymogram analyses. Enzymatic activity was investigated over a range of pH and temperature values in the culture supernatants (crude enzyme preparation). The effect of metal ions, most importantly Mn^2+^, on the endoglucanase activity and stability was also evaluated.

## 2. Materials and Methods

### 2.1. Microorganism Screening, Preservation, and Cultivation

*Streptomyces misionensis* PESB-25 was collected from a sugarcane crop soil in the State of Pernambuco, Brazil. The dilution plate technique was used for the isolation of the bacterial strain, which was selected as cellulolytic via its cultivation on solid medium containing carboxymethylcellulose low viscosity (CMC_lw_) as carbon source followed by the identification of the CMC-degrading zones using the Congo red dye [11]. Spore suspensions were prepared according to Hopwood and colleagues [12] after cultivation at 28°C for 15 days in yeast extract-malt extract-agar medium [13]. Spores were maintained in 20% (v/v) glycerol at −20°C.

### 2.2. Molecular Identification of Bacterial Strain PESB-25

Genomic DNA was extracted using the method described by Kurtzman and Robnett [14]. PCR amplification of the *rrs* gene was carried out using the GoTaq Green Master Mix kit (Promega Corporation), with primers 27F [15] and 1541R [16], in a thermal cycler model Gene Amp PCR System 9700 (Applied Biosystems). Amplified fragments were purified using the Illustra GFX PCR DNA and Gel Band Purification kit (GE Healthcare) and sequenced directly using ABI Prism dye terminator cycle sequencing reaction kit (Applied Biosystems) in an automatic sequencer (ABI model 3730; Applied Biosystems). The sequence of *rrs* gene obtained was compared with sequences online at the Ribosomal Database Project (RDP) release 10 [17] and GenBank [18] using the NCBI (The National Center for Biotechnology Information) basic local alignment search tool, BLAST (http://blast.ncbi.nlm.nih.gov/Blast.cgi) [19]. 

### 2.3. Endoglucanase Production Using Experimental Design

*Streptomyces misionensis* PESB-25 was cultivated in liquid medium with SCB and CSL as the main carbon and nitrogen sources, respectively. SCB consists of 43.8% cellulose, 25.8% hemicellulose, 22.1% lignin, 6.1% extractives, and 1.4% ash [20]. It contains, approximately, 45.3% carbon and 0.5% nitrogen [21]. CSL is a major by-product of the corn wet-milling industry and contains 47% crude protein, 26% lactic acid, 7.8% phytic acid, 2.5% reducing sugars (as dextrose), and 17% ash, total nitrogen being 7.5% [22].

Response surface methodology (RSM) was used as a tool for the optimization of SCB and CSL concentrations (independent variables) in the range indicated in Table 1. Endoglucanase activity (U·mL^−1^) was the dependent variable. A 2^2^ central composite rotational design (CCRD) was used to design experiments. 

Cultivations were carried out in 125 mL Erlenmeyer flasks containing 25 mL of mineral salts solution [23] (in g·L^−1^: NaCl, 2.0; KH_2_PO_4_, 3.0; K_2_HPO_4_, 6.0; MgSO_4_·7H_2_O, 0.5; CaCl_2_, 0.05), supplemented with a trace element solution [13] (in g·L^−1^: CuSO_4_·5H_2_O, 6.4; ZnSO_4_·7H_2_O, 1.5; FeSO_4_·7H_2_O, 1.1; MnCl_2_·4H_2_O, 7.9), with SCB and CSL at the relevant concentrations. The medium start pH was adjusted to 7.0. The growth medium was inoculated with 25 *μ*L of a spore suspension (10^9^ spores·mL^−1^) and incubated at 28°C, under agitation (200 rpm), for 3 days. The cultures were filtered through glass microfiber filter (Millipore), and the culture supernatant (crude enzyme preparation) was used for endoglucanase activity determination. 

### 2.4. Standard Endoglucanase Activity Assay

Endoglucanase activity was determined by measuring the release of reducing sugars in a reaction mixture containing 0.5 mL of the crude enzyme preparation and 0.5 mL of CMC_lw_ (SIGMA) 4.0% (w/v) solution in sodium citrate buffer 50 mM (pH 4.8) incubated at 50°C for 10 min. Reducing sugars were assayed by the dinitrosalicylic acid method [24]. One unit (IU) of endoglucanase activity corresponded to the formation of 1 *μ*mol of reducing sugars equivalent per minute under the assay conditions [25]. 

### 2.5. Effect of pH, Temperature, and Ions on the Enzyme Activity and Stability

To study the effect of pH and temperature on the supernatants endoglucanase activity, a CCRD 2^2^ was used. In the 12 experiments which were carried out, the temperature ranged from 40° to 70°C and the pH values from 3.0 to 7.0 as shown in Table 3. Citrate buffer (50 mM) was used for pH 3.0, 3.6 and 5.0 and phosphate (50 mM) for pH 6.4 and 7.0 [26]. Statistical analysis of the results was performed using the software Design Expert 7.0 (trial version), and response surface graphics were plotted with STATISTICA 7.0 (trial version).

The influence of sodium, calcium, potassium, and barium ions in the chloride form and copper, magnesium, cobalt, manganese, and iron in the sulfate form on the endoglucanase activity was done by the addition of the relevant salts at 2 mM final concentration in the enzyme activity assay using the previously determined optimal conditions for pH and temperature. The effect of Mn^2+^ was studied using at final concentrations of 1, 2, 4, 8, and 10 mM.

Endoglucanase thermal stability was evaluated at 65°C and 50°C upon incubation at different time intervals. Stability experiments were also performed in the presence of MnSO_4_ (8 mM or 16 mM) in mixtures with 1.5 mL of the crude enzyme plus 1.5 mL of MnSO_4_ solutions. In all cases, residual enzymatic activity was assayed at optimal conditions for pH and temperature, taking into account the relevant enzyme dilutions.

### 2.6. Zymogram of Endoglucanase Activity

The culture supernatants from optimized growth conditions were analyzed by electrophoresis on denaturing 10% sodium dodecyl sulphate (SDS)-polyacrylamide gel added of copolymerized CMC_lw_ (SIGMA) 0.2% (w/v) as the zymogram substrate. Electrophoresis was performed at constant voltage (100 V) at 4°C for 3 h followed by incubation with Triton X-100 sodium acetate 1.0% buffer for 30 min in ice bath for SDS removal. The detection of protein bands with endoglucanase activity was performed by incubating gels at 50°C and pH 4.8 (sodium citrate buffer 50 mM) for 30 min, followed by the gel immersion in Congo red 0.1% (w/v) for 10 min and washing with NaCl 1 M until the visualization of the enzyme bands [27]. The molecular masses of the enzyme bands seen in gels were estimated by comparing their position in the gel with a molecular mass ladder using standard molecular masses ranging from 12 to 225 kDa (Full-Range Rainbow-GE Healthcare), which was run along with the sample and photographed before Congo red staining.

## 3. Results and Discussion

The sequencing of *rrs* gene resulted in a 1491 base sequence which was 100% similar to *Streptomyces misionensis* Type Strain NRRL B-3230, and as such PESB-25 was putatively identified as a strain belonging to *S. misionensis*. The sequence obtained was submitted to the GenBank database (GenBank ID: JN869290). *S. misionensis* Type Strain NRRL B-3230 was isolated in Misiones, Argentina, and it produces misionin, an antibiotic active against phytopathogenic fungi, including *Helminthosporium* and *Alternaria* [28]. Strains from this species have been cited in the literature confirming their presence in certain soils [29] and their antibiotic production capacity [30]; however, there have been no reports that strains of this species can be cellulolytic.

**Figure 1:**
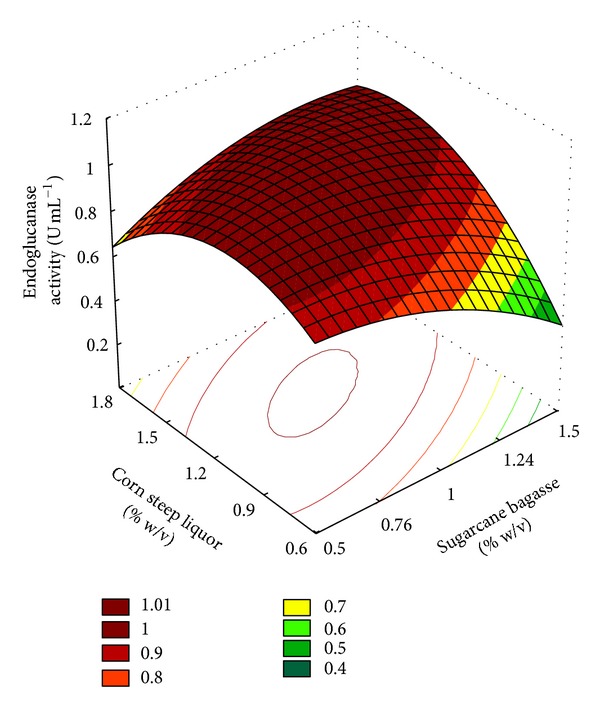
Response surface on endoglucanase production by *Streptomyces misionensis *PESB-25 using SCB and CSL concentrations as the independent variables.

The use of RSM and CCRD tools for the optimization of *Streptomyces misionensis* endoglucanase production resulted in enzyme activity accumulation in the range of 0.67 to 1.03 U·mL^−1^ (Table 1). The fitted response surface for the production of endoglucanase is given in Figure 1. Best results were obtained at center-point conditions, with SCB 1.0% (w/v) and CSL 1.2% (w/v), although results obtained in some other concentrations were not so different (e.g., 1.35% SCB and 1.03% CSL). The interaction effect evident between SCB and CSL could be related to the C : N proportion necessary for microbial growth, and consequently better enzyme production. The relevant regression equations, resulting from the analysis of variance (ANOVA) (Table 2) have shown endoglucanase production as a function of the codified values of SCB and CSL. The equation that represented a suitable model for endoglucanase production (*Y*) is given in:

(1) Y=  1.01−0.03∗  SCB−0.06∗SCB2+0.06∗CSL −  0.11∗CSL2+0.09∗SCB∗CSL±0.024.

The model *F* value of 11.74 implies that the model is significant at a high confidence level. The probability *P* value was also very low (<0.1) indicating the significance of the model. The lack of fit term was insignificant at (*α* = 0.1).

The validation of the mathematical model used was performed in triplicates and confirmed the maximal values for endoglucanase activity obtained, from 0.9 to 1.04 U·mL^−1^ when SCB 1.0% (w/v) + CSL 1.2% (w/v) were used. 

**Figure 2:**
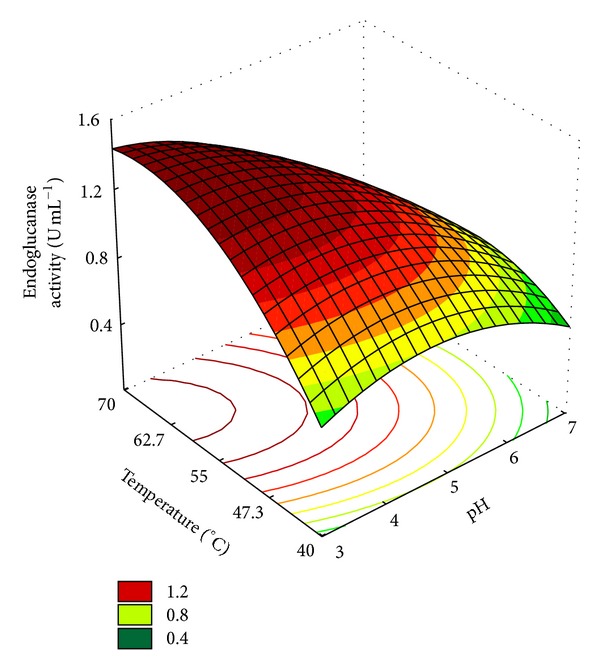
Response surface for *Streptomyces misionensis *PESB-25 endoglucanase activity by using pH and temperature values as the independent variables.

According to the data presented in Table 3, maximum endoglucanase accumulation of 1.54 U·mL^−1^ was observed at 66°C and pH 3.6. As expected, the enzyme levels were influenced by pH and temperature. Acidic conditions as well as higher temperatures favored endoglucanase activity. The analysis of the resulting surface response plots revealed that the maximal endoglucanase activity occurred in pH range of 3.0–3.6 and temperature of 62.5–70°C (Figure 2). 

The model was tested for adequacy by ANOVA (Table 4). The model *F* value of 19.67 indicates that the model is significant at a high confidence level. The probability *P* value was also very low (<0.1) indicating the significance of the model. The coefficient of determination obtained (*R* ^2^ = 0.895) indicates that 89.5% of the variability of the responses can be explained by the model. 

The regression equations, obtained after the ANOVA, demonstrated endoglucanase activity as a function of the codified values of pH and temperature. The equation that represented a suitable model for endoglucanase activity (*Y*) is given in:

(2) Y=  1.23−0.21∗pH−0.11∗pH2+0.11∗T −  0.14∗T2−0.12∗pH∗T±0.04.

The graphic of response surface (Figure 2) suggests that other ranges should be studied, so a new CCRD was performed using new pH and temperature ranges (from 2.0 to 5.0 and 55°C to 85°C), but the results were not an improvement. In this new matrix, the maximal endoglucanase activity was 1.30 U·mL^−1^ at pH 4.6 and 66°C. In this case, the best temperature was the same as before, but the enzyme activity had decreased. 

Based on the first CCRD experiment, a validation of the model was performed, using the best concentrations of C and N sources for enzymatic production [(SCB 1.0% (w/v) and CSL 1.2% (w/v)] and one of the pH and *T* conditions suggested by model, pH 3.0 and 70°C, in triplicate. The results obtained were 1.54 ± 0.01 U·mL^−1^ of endoglucanase activity that represented an increase of 50% in endoglucanase activity in comparison to that observed at pH 4.8 and 50°C. Based on these results, we can conclude that *Streptomyces misionensis* PESB-25 produces a thermoacidophilic endoglucanase. 

Cellulases with maximum activity at the acidic pH range are often observed for fungal enzymes [31] as well as for *Streptomyces*. As such, endoglucanase produced by *S. malaysiensis* AMT-3, *S. viridobrunneus* SCPE-09, *S. drozdowiczii* M7A, and *Streptomyces* sp. J2 presented maximal activity in the pH range from 4.0 to 6.0 [8–10, 32]. However, optimum pH for the *Streptomyces misionensis* PESB-25 endoglucanase was determined as 3.0, which is noteworthy. 

In general, the optimum temperature for endoglucanase activity for *Streptomyces* strains is around 50°C [8–10, 33]. Our strain showed maximum activity at 70°C, a characteristic that differs from most other *Streptomyces*. Jaradat et al. [32] described an optimal endoglucanase activity at 60°C, obtained from *Streptomyces* sp. J2, but as far as we are aware, there are no reports in the literature of an endoglucanase *Streptomyces* origin with optimal activity at such a high temperature. These unusual results concerning pH and temperature make our strain a very promising candidate for biotechnological applications, especially when very acidic and thermophilic conditions will be necessary. 

Metal ions may be a requirement for enzymatic activity and might even be an integral component of the enzyme complex [34]. Ions may also be required as cofactors for their maximum activity [35]. According to Chauvaux et al. [36], manganese and other metal ions can enhance the substrate binding affinity of the enzyme and stabilize the conformation of the catalytic site. The results for the effect of several metal ions on endoglucanase activity of *S. misionensis* PESB-25 are shown in Table 5. None of the ions studied inhibited the enzyme activity at a concentration of 2 mM. The addition of Ba^2+^ resulted in a small increase in activity (9.3%), which differs from the results reported by Grigorevski-Lima and colleagues [10], who showed that endoglucanase activity of *S. drozdowiczii* M7A greatly increased (86%) in the presence of Ba^2+^. In these experiments, the addition of Mn^2+^ and Co^2+^ to the *S. misionensis* PESB-25 supernatant resulted in significant increases in endoglucanase activity (101.5 and 61.2%, resp.). 

Considering the significant effect of Mn^2+^ 2 mM on endoglucanase activity, this effect was further evaluated. The results are shown in Table 6 and they show the effect of Mn^2+^ in the concentration range of 1 to 10 mM. This ion had a hyperactivating effect on endoglucanase, with maximum activity of 4.34 U·mL^−1^ observed with Mn^2+^ 8 mM which corresponded to an increase of 143% in endoglucanase activity in relation to when no Mn^2+^ was added. 

Although studies dealing with the activation of cellulase activity by manganese in *Streptomyces* strains have not been previously reported, there is a report on the positive effect of this ion on *Bacillus subtilis* cellulase 5A [37]. Also some fungal cellulases are activated by Mn^2+^. Gao et al. [38] studied the influence of several metal ions on activity of a purified endoglucanases from *Aspergillus terreus* and found an increase of 43% when using Mn^2+^ 2 mM. Tao et al. [39], studying *Aspergillus glaucus*, found increments of 30% when the final concentration of Mn^2+^ 4 mM was used for a purified endoglucanases obtained when growing the fungus in SCB medium. Manganese was also able to increase enzymatic activity of other enzymes, such as endonucleases from *Penicillium chrysogenum* PCL501, where an increase of 219.6% in presence of Mn^2+^ 2 mM was observed [34]. 

Few articles have been published describing cellulase production by actinomycetes using agroindustrial residues as substrates, and most of them have given very low values for endoglucanase activity when using wheat straw (WS) [40, 41] or wheat bran (WB) [10] as the main substrate. Our group has obtained values of 0.71 U·mL^−1^ when using brewer spent grain (BSG) [8], and more recently 2.00 U·mL^−1^ when using wheat bran [9]. Values as high as 4.34 U·mL^−1^, obtained in the present research, have not been described yet for endoglucanase production by actinomycetes using low-cost residues, especially SCB. 

**Figure 3:**
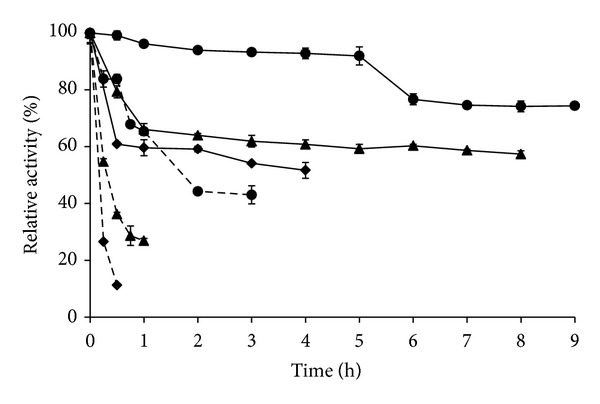
Thermal stability of *Streptomyces misionensis *PESB-25 endoglucanase activity at 65°C (- -) and 50°C (—): crude extract (filled diamond), crude extract + MnSO_4_ 8 mM (filled triangle), and crude extract + MnSO_4_ 16 mM (filled circle). Residual activity is expressed as a percentage of the original activity. Error bars represent one standard deviation of each experimental point (*n* = 3).

The results of the endoglucanase thermal stability are shown in Figure 3. When the enzyme crude extract was incubated at 65°C, the enzyme activity decreased 70% of its initial activity within 15 min of incubation. However, upon incubation at 50°C, activity decreased to 40% within 30 min, retaining this activity for 2 h. The enzyme half-life at 50°C was 4 h. 

It is known that metal ions play an important role in stabilizing proteins, protecting against thermal denaturation by binding at specific sites [36, 42]. Several studies have shown increased enzyme thermal stability in presence of calcium [27, 42, 43], which is known to regulate the stability and reactivity of a wide variety of biological proteins [43]. Given the strong positive effect of Mn^2+^ on endoglucanase activity, the effect of this ion on the enzyme stability was further investigated. It was observed that in the presence of Mn^2+^ 8 mM, the crude enzyme preparation increased 25% of its initial activity upon incubation for 30 min at 65°C, and when Mn^2+^ 16 mM was used, the activity increased to over 70%. Moreover, at a manganese ion concentration of 16 mM, the enzyme half-life at 65°C was almost 2 hours.

Results from enzyme stability at 50°C were even more promising. The incubation of crude extract with Mn^2+^ at final concentration of 16 mM resulted in an increase in thermal stability of 40% after 4 hours incubation (Figure 3), in comparison to the results for the experiments in the absence of the ion. Activity retention of over 92% for 5 h, and over 70% after 9 h of incubation, shows beyond doubt the positive effect of Mn^2+^ 16 mM on the enzyme structural stabilization. According to the overall results, incubation of the crude extract with Mn^2+^ at 50°C increased the half-life of the enzyme from 4 h (no Mn^2+^ addition) to more than 8 h (addition of Mn^2+^ 8 mM) or even more than 30 h (addition of Mn^2+^ 16 mM). Values of half-lives of 8 h have been currently reported in the literature for *Streptomyces* strains [9, 10]. 

 These are very promising results for the *Streptomyces misionensis* endoglucanase. Its natural thermal stability (which can be significantly enhanced with manganese) indicates potential as a biocatalyst for industrial process that demands long processing times at elevated temperatures, such as those in the food, sugar, and fuel ethanol industries [33]. Also, additional studies for the determination of its stability at different pH values and different periods of time would be interesting for future industrial applications. 

**Figure 4:**
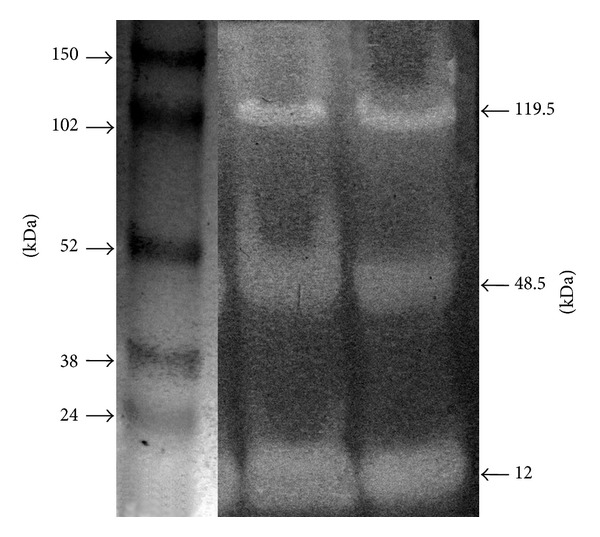
Zymogram analysis (duplicate experiment) of the culture supernatant of *S. misionensis* PESB-25 showing three bands with endoglucanase activity. Cells were grown on SCB 1.0% (w/v) and CSL 1.2% (w/v). The calculated molecular masses (in kDa) of the endoglucanases are indicated on the right side of the figure. The molecular masses of the markers Full-Range Rainbow (GE-RPN 800E) are indicated on the left side of the figure. For details see text.

The zymogram analysis of the culture supernatant of *Streptomyces misionensis* PESB-25 is shown in Figure 4. Three protein bands with endoglucanase activity and estimated molecular masses of 12.0, 48.5 and 119.5 kDa are clearly shown. Cellulose degrading microorganisms commonly produce multienzyme systems [44]. As such, and in accordance to previous reports, Nascimento and colleagues [8] observed three cellulolytic bands (51, 115, and 178 kDa) in the supernatants of *S. malaysiensis* AMT-3 when BSG 0.5% (w/v) and CSL 1.2% (w/v) were used. Da Vinha et al. [9], in their study, cultured *Streptomyces viridobrunneus* SCPE-09 in 2.0% wheat bran (w/v) and 0.19% CSL (w/v). In these conditions, two bands of endoglucanase activity were observed, one with estimated molecular masses of 37 and the other with 119 kDa. 

Additional studies about these enzymes are required to better evaluate their feasibility for further industrial applications. Purification would enable kinetics studies and also the determination of their specific activity.

## 4. Conclusions

In this study, *S. misionensis* PESB-25 was able to grow and produce endoglucanase in a culture medium containing a salt solution and agroindustrial by-products, specifically sugarcane bagasse and corn steep liquor, as the main carbon and nitrogen substrates. Characterization of the crude enzyme showed that the endoglucanases produced were acidic, thermophilic, and thermotolerant. An optimum pH of 3.0 was reported which is rare. An optimum activity temperature at 70°C was seen and is novel for actinobacterial strains. The activity of these endoglucanases was also strongly increased and more stable in the presence of a number of metal ions, especially Mn^2+^. Activity of 4.34 U·mL^−1^ was obtained under these conditions. This level of activity places this study amongst the highest described in the literature for cellulase production by *Streptomyces* strains using low-cost residues as substrates. The effect of Mn^+2^ 16 mM on enzyme stability was also important and noteworthy. Manganese at that concentration increased the enzyme stability half-life from less than 4 h to greater than 30 h at 50°C and from less than 30 minutes to 2 h at 65°C. 

The characteristics of thermoacidophiles, thermal stability, and induction by manganese suggest that endoglucanases from *S. misionensis* PESB-25 could be considered as promising alternatives in biotechnological applications. For example they could be used as a complement to fungal enzymatic mixtures improving the lignocellulose hydrolysis for ethanol production. Combining advantageous enzyme characteristics with the use of low-cost residues (SCB and CSL), we have the potential for a new low-cost enzyme production process.

## Figures and Tables

**Table 1:** Observed and predicted values of endoglucanase activity for the independent variables SCB and CSL concentrations used in central composite rotational design (CCRD), from the crude enzyme extract of *Streptomyces misionensis* PESB-25.

Run	SCB (%w/v)/Coded level	CSL (%w/v)/Coded level	Endoglucanase activity (U·mL^−1^) Observed*	Endoglucanase activity (U·mL^−1^) Predicted
1	0.65 (−1)	0.77 (−1)	0.95 ± 0.11	0.91
2	1.35 (+1)	0.77 (−1)	0.72 ± 0.005	0.67
3	0.65 (−1)	1.63 (+1)	0.86 ± 0.003	0.85
4	1.35 (+1)	1.63 (+1)	0.98 ± 0.038	0.96
5	0.5 (−1.41)	1.2 (0)	0.93 ± 0.022	0.95
6	1.5 (1.41)	1.2 (0)	0.83 ± 0.032	0.86
7	1.0 (0)	0.6 (−1.41)	0.66 ± 0.003	0.71
8	1.0 (0)	1.8 (1.41)	0.87 ± 0.024	0.88
9	1.0 (0)	1.2 (0)	1.03 ± 0.016	1.01
10	1.0 (0)	1.2 (0)	1.03 ± 0.044	1.01
11	1.0 (0)	1.2 (0)	1.00 ± 0.003	1.01

The statistical analysis of the results was performed using the software Design Expert 7.0 (trial version).

*Values are based on Mean ± SD of 3 individual observations.

**Table 2:** Statistical ANOVA for the model of endoglucanase production at different levels of concentrations of SCB and CSL.

Source of variations	Sum of squares	Degrees of freedom	Mean square	*F* value	*P* value (prob > *F*)^a^
Model	0.13	5	0.03	11.74	0.01
Residual	0.01	5	0.002		
Lack of fit	0.01	3	0.003	2.08	0.34
Pure error	0.003	2	0.001		

Total	0.14	10			

^a^Statistically significant at 90% of confidence level; *R* ^2^ = 0.84.

**Table 3:** Observed and predicted values of endoglucanase activity for the independent variables pH and temperature used in CCRD, from the crude enzyme preparation of *Streptomyces misionensis* PESB-25.

Run	pH/Coded level	Temperature (°C)/Coded level	Endoglucanase activity (U·mL^−1^) Observed	Endoglucanase activity (U·mL^−1^) Predicted
1	3.6 (−1)	44 (−1)	1.02	0.96
2	6.4 (+1)	44 (−1)	0.77	0.78
3	3.6 (−1)	66 (+1)	1.53	1.42
4	6.4 (+1)	66 (+1)	0.81	0.77
5	3.0 (−1.41)	55 (0)	1.21	1.31
6	7.0 (1.41)	55 (0)	0.72	0.72
7	5.0 (0)	40 (−1.41)	0.78	0.79
8	5.0 (0)	70 (1.41)	1.02	1.11
9	5.0 (0)	55 (0)	1.27	1.23
10	5.0 (0)	55 (0)	1.26	1.23
11	5.0 (0)	55 (0)	1.18	1.23
12	5.0 (0)	55 (0)	1.20	1.23

**Table 4:** Statistical ANOVA for the model of endoglucanase activity at different levels of pH and temperature values.

Source of variation	Sum of squares	Degrees of freedom	Mean square	*F* value	*P* value (prob > *F*)^b^
Model	0.67	5	0.13	19.67	0.001
Residual	0.04	6	0.01		
Lack of Fit	0.03	3	0.012	6.44	0.08
Pure Error	0.005	3	0.002		

Total	0.71	11			

^b^Statistically significant at 95% of confidence level; *R* ^2^ = 0.89.

**Table 5:** Effect of metal ions on endoglucanase activity. Enzyme was produced by *S. misionensis* PESB-25 grown on 1.0% (w/v) SCB and 1.2% (w/v) CSL.

Ion^a^	Relative activity (%)*	Endoglucanase activity (U·mL^−1^)
Control (no addition)	100.0	1.72
NaCl	133.2 ± 2.0	2.23
CuSO_4_	140.6 ± 0.3	2.30
MgSO_4_	126.6 ± 0.9	2.18
CoSO_4_	161.2 ± 0.6	2.73
MnSO_4_	201.5 ± 0.1	3.48
FeSO_4_	131.1 ± 0.1	2.34
CaCl_2_	137.6 ± 2.3	2.25
KCl	125.3 ± 4.9	2.17
BaCl_2_	109.3 ± 0.9	1.97

^a^The final concentration in the reaction mixture was 2 mM.

*Values are based on Mean ± SD of 3 individual observations.

**Table 6:** Effect of different manganese concentrations on endoglucanase activity.

Mn^2+^ concentration^a^	Relative activity (%)*	Endoglucanase activity (U·mL^−1^)
Control (no addition)	100.0	1.72
1 mM	182.2 ± 1.5	3.08
2 mM	201.5 ± 0.08	3.48
4 mM	185.4 ± 9.0	3.28
8 mM	243.0 ± 5.7	4.34
10 mM	233.7 ± 1.6	3.96

^a^Final Concentration in the reaction mixture.

*Values are based on Mean ± SD of 3 individual observations.

## Abbreviations

ANOVA: Analysis of variance
BLAST: Basic local alignment search tool
BSG: Brewer spent grain
CCRD: Central composite rotational design
CMC_lw_: Carboxymethylcellulose low viscosity
CSL: Corn steep liquor
NCBI: National center for biotechnology information
RDP: Ribosomal database project
RSM: Response surface methodology
SCB: Sugarcane bagasse
SDS: Sodium dodecyl sulphate
WB: Wheat bran
WS: Wheat straw.
